# Apoplastic invasion patterns triggering plant immunity: plasma membrane sensing at the frontline

**DOI:** 10.1111/mpp.12857

**Published:** 2019-07-28

**Authors:** Romain Schellenberger, Matthieu Touchard, Christophe Clément, Fabienne Baillieul, Sylvain Cordelier, Jérôme Crouzet, Stéphan Dorey

**Affiliations:** ^1^ University of Reims Champagne‐Ardenne RIBP EA 4707, SFR Condorcet FR CNRS 3417 Reims 51100 France

**Keywords:** invasion patterns, lipids, pattern‐triggered immunity, plasma membrane, PRR

## Abstract

Plants are able to effectively cope with invading pathogens by activating an immune response based on the detection of invasion patterns (IPs) originating from the pathogen or released by the plant after infection. At a first level, this perception takes place at the plasma membrane through cell surface immune receptors and although the involvement of proteinaceous pattern recognition receptors (PRRs) is well established, increasing data are also pointing out the role of membrane lipids in the sensing of IPs. In this review, we discuss the evolution of various conceptual models describing plant immunity and present an overview of well‐characterized IPs from different natures and origins. We summarize the current knowledge on how they are perceived by plants at the plasma membrane, highlighting the increasingly apparent diversity of sentinel‐related systems in plants.

## Introduction

At the base of the food web, plants are targeted by a wide range of organisms belonging to multiple branches of the evolutionary tree. To counter pathogen attack, plants use two distinct mechanisms: (i) constitutive defences, including pre‐existing physical and chemical barriers, and (ii) inducible defences activated after perception of the invader (Heath, 2000; Lee *et al.*, 2017; Veronese *et al.*, 2003).

In the 2000s, a general concept was proposed to describe plant immunity as a ‘zigzag model’ (Jones and Dangl, 2006). In this model, the recognition by the plant of pathogen‐ or microbial‐associated molecular patterns (PAMPs or MAMPs) that are typically essential components of whole classes of pathogens/microorganisms, results in PAMP‐triggered immunity (PTI). The concept of PTI was then extended to molecules that may arise from the plant itself because of the damage caused by microbes and called damage‐associated molecular patterns (DAMPs) (Boller and Felix, 2009). PTI activation classically involves cell surface‐localized pattern recognition receptors (PRRs), including membrane receptor kinases (RKs) or receptor‐like proteins (RLPs) (Boutrot and Zipfel, 2017). To proliferate, pathogens have developed the capacity to block PTI (now also called pattern‐ or PRR‐triggered immunity) by secreting effectors that interfere with perception or immunity‐related signaling (Białas *et al.*, 2018; Varden *et al.*, 2017). In the zigzag model, resistant plants are able to directly or indirectly sense effectors through intracellular nucleotide‐binding domain and leucine‐rich repeat (LRR) receptors (NLRs; a.k.a. nucleotide binding and oligomerization domain (NOD)‐like receptors) (Adachi *et al.*, 2019; Zhang *et al.*, 2017). Sensing of effectors by NLRs leads to NLR‐triggered immunity (NTI; a.k.a. effector‐triggered immunity (ETI) (Boutrot and Zipfel, 2017). Pathogens may then evolve new effector(s) able to suppress NTI and plants can evolve new NLRs in order to counter pathogen infection. Although this zigzag model is mainly valid and still largely used, it does not take into account some cases of plant immunity, suggesting that there may be no clear distinction between PTI and NTI or MAMPs/DAMPs and effectors. For instance, some apoplastic avirulence (Avr) proteins secreted by microorganisms and also considered as effectors are recognized by PRRs (de Wit, 2016b). To address the limitations and inconsistences of the zigzag model, alternative concepts of plant immunity have been proposed recently and are based on the recognition of microbial invasion via invasion patterns (IPs) that include all microbe‐derived or plant‐derived molecules (Cook *et al.*, 2015; Kanyuka and Rudd, 2019). IPs therefore encompass MAMPs, effectors (both apoplastic and cytosolic) and DAMPs, as well as any other microbe‐derived or plant‐derived evolutionary conserved or variable molecules that signal the pathogen invasion and trigger an immune response (Kanyuka and Rudd, 2019). In these models, IPs are sensed by IP receptors (IPRs). In the most recent model, IPRs are divided either in cell surface immune receptors (CSIRs) synonymous to PRRs that include RKs and RLPs or intracellular immune receptors (IIRs), mainly synonymous to NLRs (Kanyuka and Rudd, 2019). In the present review, to avoid any ambiguity, we will therefore use the general term of IPs that encompasses all molecules perceived by the plant as a danger without distinction of their origin or their potential function.

Although IP sensing at the plasma membrane through proteinaceous PRRs is well documented, an increasing number of studies has highlighted a key role of membrane lipids in the direct or indirect recognition of some IPs by plant cells. Indeed, several molecules such as necrosis and ethylene‐inducing peptide 1‐like (NLP) proteins, harpins, rhamnolipids and lipopeptides trigger plant immunity through lipid receptors and/or potential membrane lipid‐raft structure perturbations (Farace *et al.*, 2015; Henry *et al.*, 2011; Klemptner *et al.*, 2014; Sanchez *et al.*, 2012). Moreover, several synthetic compounds that mimic IP‐triggered plant signalling or perception are known to interact with biological or biomimetic lipid‐based membranes and trigger plant immunity (Bektas and Eulgem, 2015; Luzuriaga‐Loaiza *et al.*, 2018; Nasir *et al.*, 2017).

In this context, the main goal of this review is to present an overview of well‐characterized apoplastic IPs (either microbe‐ or plant‐derived) and summarize the current knowledge on their mode of perception at the plasma membrane through proteinaceous PRRs and/or lipid‐driven processes.

### IPs sensed by PRRs or involving known RK co‐receptors

**Figure 1:**
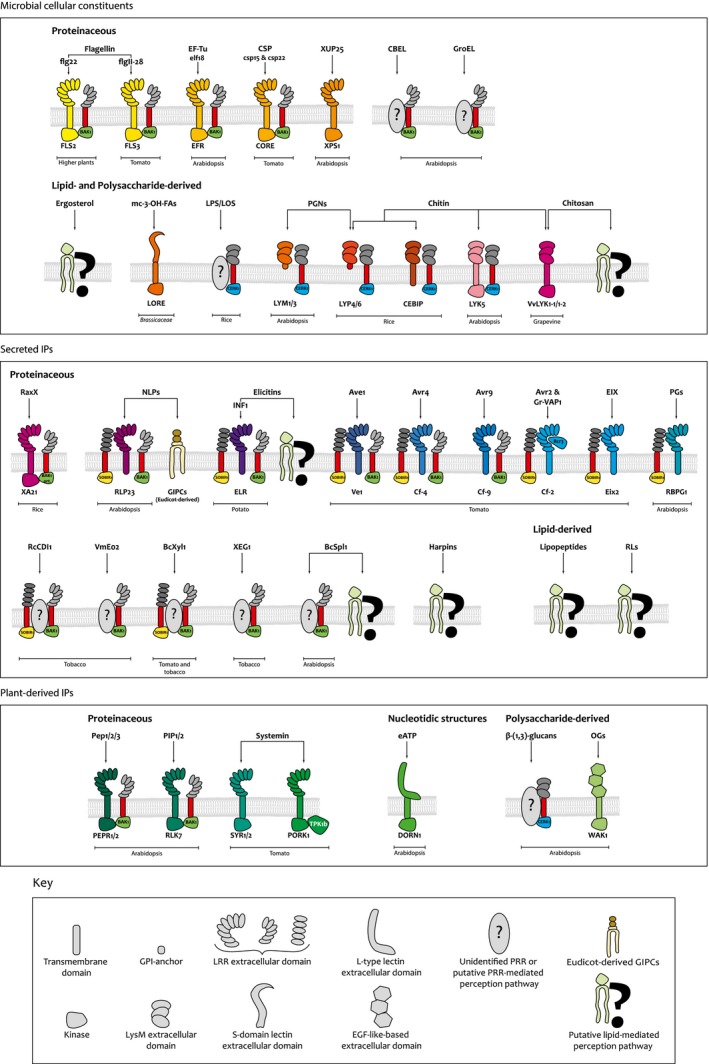
Representation of invasion pattern (IP) perception through known or potential pattern recognition receptors (PRRs) or involving plasma membrane lipids. CBEL, cellulose binding elicitor lectin; CSP, cold shock protein; EIX, ethylene‐inducing xylanase; GIPC, glycosyl inositol phosphoryl ceramide; GPI, glycophosphatidylinositol; LOS, lipooligosaccharides; LPS, lipopolysaccharides; LRR, leucine‐rich repeat; NLP, necrosis and ethylene‐inducing peptide 1‐like; OGs, oligogalacturonides; ort., orthologue; PGNs, peptidoglycans; PGs, endopolygalacturonases; RLs, rhamnolipids.

Apoplastic IPs display a large diversity of biochemical nature, including (glyco)protein‐, polysaccharide‐ and lipid‐based structures (summarized in Table 1). Most of these IPs characterized to date are known to be sensed by PRRs. For the majority of these IP/PRR couples, evidence for direct binding has been given through biochemical experiments. Proteinaceous PRRs are generally composed of an extracellular domain that binds the IP, a transmembrane domain and for some of them an intracellular kinase domain. A first distinction between PRRs can be made based on the nature of the extracellular domain such as LRR‐, LysM‐, Lectin‐, EGF‐like‐based domains (Fig. 1). A second distinction among PRRs can be made between RKs that display an intracellular kinase domain and RLPs without a kinase domain. In addition, IP perception through PRR activation has been linked to RK co‐receptors involved in signal transduction. The RK co‐receptors BAK1, SOBIR1 (both with an extracellular LRR‐type domain) and CERK1 (with an extracellular LysM‐type domain) are the best known and are major modulators of PTI (Couto and Zipfel, 2016) (Fig. 1).

**Table 1:** Examples of apoplastic invasion patterns (IPs) with known or putative perception systems in plants

IPs	Perception	Phylogenetic origin	Cellular origin	References
**IPs sensed by PRRs or involving known RKs**				
***Protein‐derived IPs sensed by LRR‐RK‐type PRRs***				
Flg22	LRR‐RK FLS2	Bacteria	Flagella	Chinchilla *et al*. (2006); Gómez‐Gómez and Boller (2000); Gómez‐Gómez *et al*. (1999)
FlgII‐28	LRR‐RK FLS3	Bacteria	Flagella	Hind *et al*. (2016)
Elf18	LRR‐RK EFR	Bacteria	Cytoplasm, secretome, cell surface	Kunze *et al*. (2004); Zipfel *et al*. (2006)
CSP	LRR‐RK CORE	Bacteria	Cytoplasm	Felix and Boller, (2003); Wang *et al*. (2016), Wei *et al*. (2018)
XUP25	LRR‐RK XPS1	Bacteria	Plasma membrane	Mott *et al*. (2016)
RaxX	LRR‐RK XA21	Bacteria	Secretome	Pruitt *et al*. (2015)
***(Glyco)protein‐derived IPs sensed by RLP‐type PRRs***				
PGNs	LysM‐RLPs LYM1/LYM3 and LYP4/LYP6	Bacteria	Cell wall	Gust *et al*. (2007)
NLPs	LRR‐RLP RLP23	Bacteria, oomycete, fungi	Secretome	Albert *et al.* (2019); Böhm *et al.* (2014)
Elicitins	LRR‐RLP ELR (INF1) and S‐domain lectin RK NgRLK1 (capsicein)	Oomycete	Secretome	Du *et al*. (2015); Kim *et al*. (2010)
Ave1	LRR‐RLP Ve1	Fungi, bacteria	Secretome	de Jonge *et al*. (2012)
Avr4	LRR‐RLP Cf‐4	Fungi	Secretome	Postma *et al*. (2016)
Avr9	LRR‐RLP Cf‐9	Fungi	Secretome	Rowland *et al*. (2005)
Avr2	LRR‐RLP Cf‐2 by targeting Rcr3	Fungi	Secretome	de Wit (2016b); Rooney *et al*. (2005); Van't Klooster *et al*. (2011)
Gr‐VAP1	LRR‐RLP Cf‐2 by targeting Rcr3	Nematode	Secretome	Lozano‐Torres *et al*. (2012)
EIX	LRR‐RLP LeEix2	Fungi	Secretome	Bar *et al*. (2010); Ron and Avni (2004)
PGs	LRR‐RLP RBPG1	Fungi	Secretome	Zhang *et al*. (2014)
***Protein‐derived IPs sensed by unidentified PRRs but requiring RK co‐receptors***				
CBEL	Co‐receptor BAK1	Oomycete	Cell wall	Larroque *et al*. (2013)
GroEL	Co‐receptor BAK1	Bacteria (aphid endosymbiont)	Cytoplasm	Chaudhary *et al*. (2014)
BcSpl1	Co‐receptor BAK1	Fungi	Secretome	Frías *et al*. (2011, 2013)
RcCDI1	Co‐receptors BAK1 and SOBIR1	Fungi	Secretome	Franco‐Orozco *et al*. (2017)
VmE02	Co‐receptor BAK1	Fungi	Secretome	Nie *et al.* (2019)
BcXyl1	Co‐receptors BAK1 and SOBIR1	Fungi	Secretome	Yang *et al*. (2018)
XEG1	Co‐receptor BAK1	Oomycete	Secretome	Ma *et al*. (2015)
***Lipid‐derived and polysaccharide‐derived IPs from microbial origin***				
LPS/LOS	Co‐receptor CERK1	Bacteria	Cell wall	Desaki *et al*. (2018)
Medium‐chain 3‐hydroxy fatty acids	Bulb‐type lectin RK LORE	Bacteria	Unknown	Kutschera *et al*. (2019)
Chitin	LysM‐RLPs CEBIP and LYP4/LYP6 LysM‐RKs LYK5 and VvLYK1‐1 /VvLYK1‐2	Fungi, arthropod, oomycete	Cell wall, exoskeleton	Cao *et al*. (2014); Hayafune *et al*. (2014); Liu *et al*. (2012)
Chitosan	Potential WAK1 and GsSRK receptors LysM‐RKs VvLYK1‐1 /VvLYK1‐2	Fungi	Cell wall	Brulé *et al*. (2018); Liu *et al*. (2018)
***IPs from plant origin***				
AtPep1/2/3	LRR‐RKs PEPR1/PEPR2	Plant	Cytoplasm	Krol *et al*. (2010)
PIP1/2	LRR‐RK RLK7	Plant	Secretome	Hou *et al*. (2014)
Systemin	LRR‐RKs SYR1/SYR2 and LRR‐RK PORK1	Plant	Cytoplasm	Santamaria *et al*. (2018); Wang *et al*. (2018); Xu *et al*. (2018)
eATP	L‐type lectin RK DORN1	All reign	Cytoplasm	Choi *et al*. (2014)
β‐(1,3)‐glucans	Co‐receptor CERK1	Plant	Vacuole	Mélida *et al*. (2018)
OGs	WAK1 RK	Plant	Cell wall	Brutus *et al*. (2010)
**IPs sensed through plasma membrane lipid interaction**				
***Proteinaceous IPs***				
NLPs	GIPCs	Bacteria, oomycete, fungi	Secretome	Lenarčič *et al*. (2017)
Harpins	Lipids	Bacteria	Secretome	Choi *et al*. (2013); Lee *et al*. (2001)
Elicitins	Sterols	Oomycete	Secretome	Derevnina *et al*. (2016); Gerbeau‐Pissot *et al*. (2014)
***Lipid‐based IPs***				
Ergosterol	Lipid raft disturbance	Fungi	Plasma membrane	Rossard *et al*. (2010); Xu *et al*. (2001)
Lipopeptides	Phospholipids	Bacteria	Secretome	Henry *et al*. (2011)
RLs	Phosphatidylcholines/POPC/phosphatidylinositol/ phosphatidylglycerol/β‐sitosterol/glucosylceramide	Bacteria	Secretome	Sanchez *et al*. (2012); Monnier *et al*. (2019)
SRBs	PLPC/β‐sitosterol	Synthetic	/	Luzuriaga‐Loiaza *et al*. (2018)
Ac‐RL/Alk‐RL	PLPC/β‐sitosterol	Synthetic	/	Nasir *et al*. (2017)
***Polysaccharide‐derived structures***				
Chitosan	Phospholipids	Fungi	Cell wall	Amborabé *et al.* (2000); Rossard *et al*. (2010)

GIPC, glycosyl inositol phosphoryl ceramide (sphingolipid); PLPC, 1‐palmitoyl‐2‐linoleoyl‐sn‐glycero‐3‐phosphocholine (phospholipid); POPC, 1‐palmitoyl‐2‐oleoyl‐glycero‐3‐phosphocholine (phospholipid).

#### Protein‐derived IPs sensed by LRR‐RK‐type PRRs

Bacterial protein‐derived IPs are the most studied apoplastic plant immune‐inducing molecules. Among them, flagellin, a subunit protein of the flagellum present in many bacteria including *Pseudomonas* and *Xanthomonas* species, is the best characterized. As flagella are essential structures for bacterial fixation to host cells and motility, flagellin is an optimal target for the plant immune system. Flagellin monomers can be found in the extracellular space either during flagellum construction or due to damage to flagellar filaments (Gómez‐Gómez and Boller, 2002). It was recently demonstrated in *Nicotiana benthamiana* that plant‐secreted β‐galactosidase 1 (BGAL1) promotes hydrolytic release of the active IP from glycosylated flagellin. BGAL1 acts in immunity against pathogenic *Pseudomonas syringae* strains only when they carry a terminal modified viosamine in the flagellin *O*‐glycan. Interestingly, *P. syringae* pathovars are able to evade host immunity by using BGAL1‐resistant *O*‐glycans or by producing a BGAL1 inhibitor (Buscaill *et al*., 2019). Although flagellin is perceived by a large set of plants, the peptide sequence necessary for recognition and the cognate PRRs vary among plant species (Boller and Felix, 2009; Trdá *et al.*, 2015). For instance, a 22‐amino acid peptide (flg22), originating from the N‐terminal extremity of flagellin is perceived by FLS2, a LRR‐RK present in several monocots and eudicots (Chinchilla *et al.*, 2006; Gómez‐Gómez and Boller, 2000; Gómez‐Gómez *et al.*, 1999). In tomato, FLS3 senses another part of flagellin, flgII‐28, independently of FLS2 (Hind *et al.*, 2016).

Elf18, an 18‐amino acid‐long sequence originating from the N‐terminal part of bacterial elongation factor Tu (EF‐Tu), has also been widely studied for its ability to induce a plant immune response (Zipfel *et al.*, 2006). EF‐Tu is localized at the bacterial surface (in outer membrane vesicles) and in the secretome of several bacteria (Katsir and Bahar, 2017; Zipfel *et al.*, 2006). EF‐Tu and its elf18 peptide are perceived by the LRR‐RK EFR in *Arabidopsis thaliana* (hereafter, *Arabidopsis*)*.* Interestingly, elf18 does not trigger immunity‐related mechanisms in plants outside the Brassicaceae, unlike flg22 (Kunze *et al.*, 2004; Zipfel *et al.*, 2006). Similarly, the conserved domains of bacterial cold shock protein (CSP), csp15 and csp22, are perceived by the LRR‐RK CORE that is only present in Solanaceae (Felix and Boller, 2003; Wang *et al.*, 2016; Wei *et al.*, 2018). CSPs are intracellular (cytoplasmic) proteins and how they become available for perception by PRRs is still nebulous. XUP25 peptide, originating from *P. syringae* uracil/xanthine permease, induces immunity through the LRR‐RK XPS1 in *Arabidopsis* (Mott *et al.*, 2016). How the peptide is liberated from the main protein remains unknown. Plant proteases that participate in plant immunity could be involved in the process (Balakireva and Zamyatnin, 2018). Until now, no RK co‐receptor required for signal transduction has been associated with XPS1. It should be noted that the majority of the protein‐derived IPs originating from microbes and sensed by LRR‐RK‐type PRRs are not secreted but are microbial constituents. To date, the RaxX tyrosine‐sulphated protein that is secreted by Gram‐negative bacteria (in particular *Xanthomonas* species) and sensed by the rice LRR‐RK XA21 (Pruitt *et al.*, 2015) is the only exception. Interestingly, XA21 interacts with the rice BAK1 orthologue, OsSERK2. All these PRRs (excepted XPS1 for which it has not yet been investigated) require the co‐receptor BAK1 or an orthologue to trigger signal transduction.

#### (Glyco)protein‐derived IPs sensed by RLP‐type PRRs

The bacterial cell wall is well known to be a source of IPs. Peptidoglycans (PGNs), which provide rigidity and structure to the bacterial cell wall in both Gram‐negative and Gram‐positive bacteria, induce innate immunity in monocots and eudicots. Muropeptides that are PGN breakdown products from *Agrobacterium* and *Xanthomonas* are sensed by *Arabidopsis* (Erbs *et al.*, 2008). In addition, *Staphylococcus aureus* PGNs mediate immune stimulation in *Arabidopsis* based on recognition of the PGN sugar backbone (Gust *et al.*, 2007). Perception of PGNs in *Arabidopsis* involves the co‐receptor CERK1 and the LysM domain RLPs LYM1 and LYM3 (Willmann *et al.*, 2011). Notably, CERK1 is also necessary for signal transduction upon chitin perception in this plant (Miya *et al.*, 2007). In rice, LYP4 and LYP6, the homologous RLPs of LYM1 and LYM3, mediate PGNs and chitin sensing by interacting with CERK1 (Cao et al., 2014; Liu *et al.*, 2012). Whereas the majority of protein‐derived IPs is perceived by LRR‐type PRRs, it is interesting to notice that PGNs are sensed by LysM‐type PRRs, clearly suggesting that it is the glycan part of the molecule that is recognized by plants. Although PGNs are present in the outer leaflet of Gram‐positive bacteria, they are localized in the periplasmic space under the outer membrane in Gram‐negative bacteria and are therefore hardly available for perception by PRRs (Silipo *et al.*, 2010). However, plants can produce apoplastic PGN hydrolases such as LYS1 that could release elicitor PGN fragments from insoluble bacterial cell walls (Liu *et al*., 2014).

Numerous protein‐derived IPs are secreted in the apoplast by pathogens. Their direct accessibility to PRRs makes them interesting targets for the plant immune system. NLPs form a family of proteins secreted by phytopathogenic bacteria, oomycetes and fungi (Gijzen and Nurnberger, 2006; Lenarčič *et al.*, 2017). These proteins, characterized by their necrosis‐inducing *Phytophthora* protein1 (NPP1) domain, induce plant immunity and cell death in eudicot plant species (Lenarčič *et al.*, 2017; Qutob *et al.*, 2006). The PRR‐mediated sensing of nlp20, a conserved 20‐mer fragment from NLPs, has been identified in *Arabidopsis* and involves the LRR‐RLP RLP23 and the SOBIR1/BAK1 complex (Albert *et al.*, 2015; Böhm *et al*., 2014). Elicitins, first characterized in the 1980s, are IPs secreted by the oomycetes *Phytophthora* sp. and *Pythium* sp. (Ricci *et al.*, 1989). They induce defence responses and a localized programmed cell death called hypersensitive response (HR) in various plant species (Derevnina *et al.*, 2016). The LRR‐RLP ELR from potato is able to sense the elicitin INF1 and associates with BAK1 and SOBIR1 to activate defence responses (Domazakis *et al.*, 2018; Du *et al.*, 2015). In tomato, elicitin perception involves the co‐receptor/adaptor kinase SOBIR1 that associates with several RLPs to induce plant immunity (Gust and Felix, 2014; Peng *et al.*, 2015). Interestingly, unlike INF1, the *Phytophthora capsici* capsicein was shown to interact *in vitro* with the S‐domain lectin RK NgRLK1 from *Nicotiana glutinosa* (Kim *et al.*, 2010).

Several IPs, often referred to as apoplastic effectors according to the zigzag model, are perceived by RLP‐type PRRs. Ave1 secreted by *Verticillium dahliae* is sensed by the RLP Ve1 in tomato (de Jonge *et al.*, 2012) and this perception requires BAK1 (Fradin *et al.*, 2009) and SOBIR1 (Liebrand *et al.*, 2013). Interestingly, Ave1 is homologous to plant natriuretic peptides (PNPs), which are mobile signalling hormones secreted in the apoplast under biotic and abiotic stresses and for which a cognate plasma membrane LRR‐RK has been characterized in *Arabidopsis* (Turek and Gehring, 2016). Avr4 and Avr9 are two other fungal proteins secreted by *Cladosporium fulvum*. They promote, respectively, Cf‐4 and Cf‐9 RLP association with BAK1 to initiate an immune response in tomato. Avr2, another *C. fulvum* effector, is recognized by the RLP Cf‐2. Cf‐4‐ and Cf‐2‐mediated immunity both require SOBIR1 as co‐receptor (Liebrand *et al.*, 2013; Postma *et al.*, 2016). Interestingly, Avr2 is also able to inhibit the apoplastic tomato cysteine protease Rcr3 and the direct interaction is necessary to trigger Cf‐2‐dependent HR and resistance to *C. fulvum* (Rooney *et al.*, 2005; Van't Klooster et al., 2011). Reminiscent of the Avr2/Rcr3/Cf‐2 mechanism of perception, the nematode venom allergen‐like protein Gr‐VAP1, involved in *Globodera rostochiensis* virulence, targets the Rcr3 protein from *Solanum pimpinellifolium* (Lozano‐Torres *et al.*, 2012).

Some phytopathogenic fungi, mostly necrotrophs, secrete cell wall‐degrading enzymes (CWDE) (Kubicek *et al.*, 2014). Some of these enzymes are directly recognized by plant cells and not through the DAMPs that could be produced by their lytic activity. This is the case of xylanases involved in hemicellulose degradation. The fungal ethylene‐inducing xylanase (EIX) is directly perceived by tomato cells through the RLP Eix2, leading to activation of defence responses and induction of an HR (Bar *et al.*, 2010; Ron and Avni, 2004). Moreover, Eix2 interacts with SOBIR1 (Liebrand *et al.*, 2013). Endopolygalacturonases (PGs) are other fungal CWDEs that degrade pectins and act as virulence factors for several fungal pathogens. In *Arabidopsis*, the LRR‐RLP RBPG1 has been identified as a receptor for BcPG2, BcPG3, BcPG4 and BcPG6 originating from *Botrytis cinerea* and *Aspergillus niger*. This recognition process involves the co‐receptor SOBIR1 (Zhang *et al.*, 2014). In grapevine, BcPG1, an endopolygalacturonase from *B. cinerea*, was shown to activate plant defence responses independently of its enzymatic activity, also suggesting a direct recognition in this plant (Poinssot *et al.*, 2003). Remarkably, all these LRR‐type RLPs lacking the cytoplasmic signalling competent moiety require the RK co‐receptors SOBIR1 and/or BAK1 for signal transduction (Liebrand *et al.*, 2014).

#### Protein‐derived IPs sensed by unidentified PRRs but requiring RK co‐receptors

Many of the previously described RK or RLP PRRs have been identified through forward or reverse genetic approaches and for most of them a direct interaction with the corresponding IP has been confirmed by receptor‐ligand binding experiments. However, for a number of IPs known to induce plant immunity, their cognate receptor is not yet identified albeit the involvement of RK co‐receptors has been highlighted.

CBEL (cellulose binding elicitor lectin), a glycoprotein from the cell wall of *Phytophthora nicotianae*, is perceived by *Arabidopsis* and tobacco. In *Arabidopsis*, BAK1 is necessary for CBEL‐mediated immunity (Larroque *et al.*, 2013). Interestingly, its cellulose‐binding domain (CBD) is sufficient to induce plant defence responses (Gaulin *et al.*, 2006). The intracellular chaperonin GroEL from the aphid endosymbiont *Buchnera aphidicola* was also shown to induce a BAK1‐dependent immune response in *Arabidopsis* (Chaudhary *et al.*, 2014). BcSpl1, an abundant cerato‐platanin protein present in the secretome of the necrotrophic fungus *B. cinerea*, triggers a BAK1‐dependent HR in tomato, tobacco and *Arabidopsis* (Frías *et al.*, 2011, 2013). Similarly, the small cysteine‐rich protein VmE02 secreted by another necrotrophic fungus *Valsa mali* and XEG1, a *Phytophthora sojae* secreted glycoside hydrolase, triggers plant defences and cell death in a BAK1‐dependent manner in tobacco (Ma *et al.*, 2015; Nie *et al.*, 2019). For some IPs, the involvement of both BAK1 and SOBIR1 in plant immunity associated with an HR has been demonstrated. This is the case for RcCDI1, a small protein with an unknown function secreted by *Rhynchosporium commune*, which is only recognized by eudicots (Franco‐Orozco *et al.*, 2017) and the *B. cinerea* xylanase BcXyl1 active on tobacco and tomato (Yang *et al.*, 2018). It should be noted that the co‐receptors BAK1 and SOBIR1 are only involved in the perception of protein‐derived IPs so far. In addition, SOBIR1 has only been linked to the perception of secreted IPs.

#### Lipid‐derived and polysaccharide‐derived IPs from microbial origin

Lipopolysaccharides (LPS) and lipooligosaccharides (LOS) are major constituents of bacterial Gram‐negative cell walls and are well known for inducing immunity in several plant species (Desender *et al.*, 2006; Dow *et al.*, 2000). LPS consist of a lipid A portion, an oligosaccharide core and an *O*‐polysaccharidic extremity. In tobacco, treatment with lipid A induces late‐phase defence responses while the oligosaccharide core induces early immune responses (Erbs and Newman, 2012; Silipo *et al.*, 2005). In *Arabidopsis*, the lipid A fragment carries the minimal molecular pattern recognized by plants (Ranf *et al.*, 2015). Recently, it was shown that the Brassicaceae specific bulb‐type lectin RK LORE recognizes medium‐chain 3‐hydroxy fatty acid (mc‐3‐OH‐FA) metabolites present in the lipid A structures from *Pseudomonas* and *Xanthomonas* bacteria (Kutschera *et al.*, 2019). mc‐3‐OH‐FAs are sensed in a chain length‐ and hydroxylation‐specific manner. Interestingly, bacterial compounds comprising mc‐3‐OH‐acyl building blocks but devoid of free mc‐3‐OH‐FAs (such as lipid A but also lipopolysaccharides, rhamnolipids, lipopeptides and acyl‐homoserine‐lactones) do not trigger LORE‐dependent immunity (Kutschera *et al.*, 2019). Recently, it has also been shown that LPS can induce LORE‐independent immunity in *Arabidopsis* (Shang‐Guan *et al.*, 2018). Interestingly, in rice the perception of LPS is partly dependent on CERK1, while this co‐receptor is not involved in LPS sensing in *Arabidopsis* (Desaki *et al.*, 2018; Ranf *et al.*, 2015). This indicates that LPS perception mechanisms in monocots and eudicots could require different receptor complexes and potentially different molecular patterns. LPS, as constituents of the cell wall, are hardly available for plant cell perception and the exact mechanisms of LPS delivery to plant cells is not fully understood. Surfactants were shown to promote the release of LPS from bacterial cells (Al‐Tahhan *et al.*, 2000) and plant lipid binding protein (LBP)‐like proteins or bacterial outer membrane vesicles could also be involved in LPS delivery (Iizasa *et al.*, 2016; Katsir and Bahar, 2017).

Plant genomes encode several hydrolytic enzymes, including chitinases and glucanases commonly known as pathogenesis‐related (PR) proteins that can use the fungal cell wall as a substrate to release chitin and glucans (Pusztahelyi, 2018). Chitin, an homopolymer of β‐(1,4)‐linked *N*‐acetyl‐d‐glucosamine (GlcNAc) present in fungi and oomycete cell wall and arthropod exoskeleton, is a widespread IP perceived by monocot and eudicot plants. Perception of chitin and chitin‐derived oligosaccharide structures by plants depends on the acetylation and/or the polymerization degree of these compounds (Pusztahelyi, 2018). Chitin perception in rice and *Arabidopsis* implies PRRs sharing a similar extracellular LysM domain (Cao *et al*., 2014). In rice, the RLP OsCEBIP binds chitin and interacts with OsCERK1 to trigger signalling events (Hayafune *et al.*, 2014). The LYP4 and LYP6 CEBiP‐like RLPs from rice are also able to bind chitin and are partially involved in its recognition by the plants (Liu *et al.*, 2012). In *Arabidopsis,* CERK1 is also necessary for chitin sensing. This LysM‐containing RK interacts with chitin and LYK5, another LysM RK, to induce defence responses (Cao *et al*., 2014). Chitosan, a modified form of chitin only found in fungi that possess deacetylase enzymes, is also perceived by several plants (Hadwiger, 2013; Zuppini *et al.*, 2004). Chitosan oligosaccharide binding proteins were recently identified from the plasma membrane of wheat leaf cell and include W5G2U8, a potential WAK1 receptor protein, and W5HY42 and W5I0R4, which are potential GsSRK (G‐type lectin S‐receptor‐like serine/threonine‐protein kinases) receptor proteins (Liu *et al.*, 2018). Whether these proteins directly interact with chitosan is still unknown. Recently, it was demonstrated that chitin and chitosan perception involves VvLYK1‐1 and VvLYK1‐2 in grapevine (Brulé *et al.*, 2018). β‐(1,6)‐glucans, important polysaccharides from fungi and oomycetes, have been extensively studied for their capacity to induce an immune response in plants. How these molecules are sensed by plants, however, is poorly understood (Fesel and Zuccaro, 2016). Remarkably, the extracellular domains of PRRs involved in the recognition of polysaccharide‐derived IPs from microbes and characterized to date are only from LysM‐types.

#### IPs from plant origin

Plants are able to detect damage caused by pathogens or pests (Gust *et al.*, 2017). Among IP endogen peptides, AtPeps are the best known. AtPep1, AtPep2 and AtPep3 are perceived by the LRR‐RKs PEPR1 and PEPR2 in *Arabidopsis* (Krol *et al.*, 2010). AtPep1 is derived from the C‐terminus part of PROPEP1 protein and is over‐expressed following wounding, cell wall degradation and IP perception (Krol *et al.*, 2010). Similarly, PIP1 and PIP2, two other pathogen‐inducible endogen peptides, are recognized by plants. After infection, prePIP1 and prePIP2 are secreted in the extracellular space and cleaved at the C‐terminus. The LRR‐RK RLK7 participates in the perception of PIP1 (Hou *et al.*, 2014).

Systemin is an 18 amino acid peptide from Solanaceae released in the apoplast by an unknown mechanism. Systemin interacts with the LRR‐RKs SYR1 and SYR2 to induce defence against insect herbivory (Wang *et al.*, 2018). PORK1, another LRR‐RK from tomato closely related to the SYR1 and SYR2 proteins, is also required for systemin‐induced defences (Xu *et al.*, 2018). Whether SYR1/2 could act in concert with PORK1 remains to be investigated. PORK1 interacts and phosphorylates the protein kinase TPK1b to induce systemin‐driven immunity. Interestingly, systemin treatment does not induce ROS production and phosphorylation cascade activation is not reduced in the PORK1 RNAi line (Xu *et al.*, 2018), suggesting that systemin also induces PORK1‐independent defence mechanisms in plants.

DORN1, a purinoreceptor from the L‐type lectin RK family, senses extracellular ATP (eATP) in *Arabidopsis* and is necessary for eATP‐mediated defence induction*.* Interestingly, it only perceives eATP and no other eNTPs (Choi *et al.*, 2014). How eATP is available for perception by the PRR remains unknown. One hypothesis could be a release by cellular lysis upon pathogen infection. Even though other nucleotidic molecules like bacterial RNAs (Lee *et al.*, 2016) and extracellular small DNA fragments (Duran‐flores and Heil, 2018) are known to induce plant immunity, only eATP was identified with a cognate PRR.

Although β‐(1,6)‐glucans are generally specific of fungi and oomycetes, β‐(1,3)‐glucans are naturally present in plant cell walls (Fesel and Zuccaro, 2016). In *Arabidopsis*, it was recently shown that non‐branched β‐(1,3)‐glucans sensing requires CERK1, the co‐receptor also involved in chitin perception (Mélida *et al.*, 2018). Laminarin, a β‐(1,3)‐glucan polymer with β‐(1,6) branches produced by *Laminaria digitata* alga, induces a PTI in several plants, including grapevine (Aziz *et al.*, 2003), tobacco (Klarzynski *et al.*, 2000) and *Arabidopsis* (Ménard *et al.*, 2004). Interestingly, a sulphated‐derived structure of this β‐(1,3)‐glucan is even more active (Menard *et al*., 2004; Trouvelot *et al.*, 2008). Oligogalacturonides (oligomers of α‐(1,4)‐linked galacturonosyl residues, OGs), released from pectin after degradation by fungal polygalacturonases, associate with WAK1, a singular EGF‐like‐RK to trigger plant immunity (Brutus *et al.*, 2010). Overall, plant protein‐derived IPs are sensed by receptors carrying an LRR domain (like microbial IPs) and more specifically belonging to the RK family. In addition, plant β‐(1,3)‐glucan recognition requires the co‐receptor CERK1, which is mainly associated with LysM‐type PRRs and is also involved in the sensing of polysaccharide‐derived IPs from microbes.

### IPs sensed through plasma membrane lipid interaction

Like proteins, lipids are major components of plasma membranes. Lipids, as the first components encountered by IPs able to bind or insert into plant plasma membranes, could participate in their initial sensing and the establishment of a ‘danger’‐related immune response. Various studies suggest that some IPs may directly interact with lipids (and not PRRs) either modulating plasma membrane physical properties (driven by insertion between lipids and/or membrane damages) or, as demonstrated more recently, using lipid decoration as the receptor/target (Mamode‐Cassim *et al.*, 2019). In all cases, this interaction of IPs with plasma membrane lipids could change the behaviour and functions of membrane microdomains/nanodomains containing a variety of integral membrane proteins, such as mechanoreceptors, ion channels, membrane receptors and enzymes. The changes in location and/or activity of membrane proteins after lipid binding could lead to immune signalling activation (Fig. 1). IPs interacting with plasma membrane lipids are often of hydrophobic or amphiphilic nature and include proteinaceous, lipid‐derived or polysaccharide‐derived structures (Table 1). Moreover, some of these compounds can be classified as or are related to toxin‐like compounds.

#### Proteinaceous IPs

The best recent example of IPs directly binding to lipids is represented by cytotoxic NLPs. NLP‐mediated phytotoxicity and plant defence gene expression are closely related, suggesting that toxin‐mediated interference with host integrity triggers plant immunity‐associated responses. This phytotoxin‐mediated activation of plant immunity is reminiscent of microbial toxin‐induced inflammasome activation in vertebrates, which results in secretion of cytokines and programmed pro‐inflammatory cell death (Ottmann *et al.*, 2009; Qutob *et al.*, 2006). Even though a 20‐mer conserved fragment from NLPs induces plant immunity through RLP23/SOBIR1/BAK1 protein complex, cytotoxic NLPs also directly bind to glycosyl inositol phosphoryl ceramide (GIPC) (Lenarčič *et al.*, 2017). GIPCs are the most abundant sphingolipids in plant membranes and comprise 60–80% lipids in the outer leaflet of the plasma membrane (Lenarčič *et al.*, 2017; Van den Ackerveken, 2017). NLPs can bind terminal monomeric hexose moieties of GIPCs, resulting in conformational changes within the protein. The NLP/GIPC binding has been quantified by surface plasmon resonance analysis with a dissociation constant of around 300 nM in *Arabidopsis*. Only eudicot plants are affected by NLPs. Insensitivity of monocot plants to NLPs may be explained by the length of the GIPC headgroup, consisting of three‐terminal hexoses instead of two for eudicots (Lenarčič *et al.*, 2017; Van den Ackerveken, 2017). NLPs are known as toxin‐like or virulence factors, therefore sphingolipids described as ‘receptors’ could also be considered as ‘targets’ for NLPs. However, it can be assumed that both the PRR recognition and the toxin‐like effects through lipid binding could act in concert to be used by the plant to perceive a ‘danger’ and to activate a strong innate immune response leading to an active plant cell death process (Qutob *et al.*, 2006). Similarly, the cerato‐platanin BcSpl1 secreted by a necrotrophic fungus could be recognized as a ‘danger’ signal via a BAK1‐dependent process and a less specific and indirect sensing through lipid‐driven perturbation. Accordingly, BcSpl1 was shown to associate with the plant plasma membrane, triggering rapid morphological changes at the cellular level (Frías *et al.*, 2014).

Harpins are proteins secreted by type III secretion systems of Gram‐negative bacteria like *Erwinia amylovora* and have been very well known as IPs since the 1990s (Baker *et al.*, 1993; Wei *et al.*, 1992). Harpins induce defence responses in several plant species (Choi *et al.*, 2013). They are able to interact with lipids to form pores in artificial membranes and they participate in virulence to several bacteria (Choi *et al.*, 2013). Interestingly, a non‐proteinaceous harpin binding site has been characterized in tobacco plasma membranes. It mediates activation of the PR gene *HIN1* through mitogen‐activated protein kinase activity, independently of extracellular calcium fluxes (Lee *et al.*, 2001). Elicitins and cryptogein in particular are known to bind sterols and other lipids with various affinities. Independent studies have revealed that elicitins can act as sterol carriers by scavenging sterols from synthetic liposomes and plant plasma membranes (Derevnina *et al.*, 2016). Elicitin‐induced cell death could be due to disruption of plant plasma membrane integrity upon interaction. Interestingly, long‐chain bases sphingolipids (LCBs) and their phosphorylated derivatives present in the plasma membrane differentially regulate cryptogein‐induced production of reactive oxygen species (ROS) in tobacco cells (Coursol *et al.*, 2015). In addition, the use of fluorescence recovery after photobleaching revealed an increase in plasma membrane fluidity induced by cryptogein, but not by flagellin (Gerbeau‐Pissot *et al.*, 2014). As for NLPs, it can be postulated that both PRR recognition and lipid‐driven perturbations could act participate in elicitin‐related strong immune responses.

#### Lipid‐based IPs

Several lipid‐derived IPs have already been discovered, including arachidonic acid, eicosapolyenoic acid (Bostock *et al.*, 1981, 2011; Robinson and Bostock, 2015) and cerebroside (Umemura *et al.*, 2002, 2004). However, little is known about their perception by plants.

Ergosterol, a fungi‐specific sterol, induces immunity‐related markers in tobacco and tomato (Klemptner *et al.*, 2014). Interestingly, this molecule triggers apoplastic medium alkalinization in tomato, unlike plant sterols (Granado *et al.*, 1995). It has been hypothesized that plants either possess an ergosterol receptor or that ergosterol uptake could lead to perturbations of lipid raft structures because of their ability to form very stable microdomains (Klemptner *et al.*, 2014; Xu *et al.*, 2001). In this respect, ergosterol directly affects *Beta vulgaris* plasma membrane H^+^‐ATPase activities, indicating that it could impact the structural organization of lipid rafts from this plant plasma membrane (Rossard *et al.*, 2010).

Cyclic lipopeptides are amphiphilic molecules produced by a large variety of bacteria such as *Streptomyces*, *Pseudomonas* and *Bacillus* (Raaijmakers *et al.*, 2010). Lipopeptides have emerged as key players in the induction of plant immunity driven by beneficial microorganisms (Falardeau *et al.*, 2013; Raaijmakers *et al.*, 2010). *Bacillus subtilis* is known to produce three main families of cyclic lipopeptides, namely surfactins, iturins and fengycins (Falardeau *et al.*, 2013). Mycosubstilin, a lipopeptide from the iturin family, plipastatin from the fengycin family and surfactin activate immunity‐related markers in grapevine, cotton and *Arabidopsis* (Debois *et al.*, 2015; Farace *et al.*, 2015; Han *et al.*, 2015). Mycosubtilin and fengycin are known to interact with membrane lipids (Deleu *et al.*, 2005, 2008; Maget‐Dana and Ptak, 1990; Nasir *et al.*, 2010). Massetolide A, secreted by *Pseudomonas fluorescens* SS101, also induces plant defence mechanisms in tomato (Tran *et al.*, 2007) and orfamide produced by *Pseudomonas* spp. has recently been shown to induce rice and bean immunity (Ma *et al.*, 2016, 2017). Surfactin studies highlighted that the lipopeptide structure strongly impacts its ability to trigger an immune response. Surfactins with C14 and C15 chain length induce extracellular medium alkalinization, unlike C12 and C13 (Jourdan *et al.*, 2009). Importantly, it was demonstrated that surfactin has to target the lipid fraction of the plant plasma membrane in order to induce immune‐related defence responses (Henry *et al.*, 2011). Longer chain length surfactins displayed stronger interactions with membranes compared to C12 and C13 variants. Moreover, there was no refractory state induced by repeated stimulations with surfactin. It was therefore proposed that surfactin perception relies on a lipid‐driven process rather than a direct sensing by high‐affinity protein receptors (Henry *et al.*, 2011). Rhamnolipids (RLs) are amphiphilic molecules secreted by *Pseudomonas* and *Burkholderia* species and involved in bacterial motility and biofilm formation (Abdel‐Mawgoud *et al.*, 2010). RLs are able to induce *Brassica napus, Arabidopsis* and *Vitis vinifera* immunity (Monnier *et al.*, 2018; Sanchez *et al.*, 2012; Varnier *et al.*, 2009). Rhamnose alone is not responsible for this immune response (Varnier *et al.*, 2009). Given their amphiphilic nature, it was postulated that RLs could interact with plant membrane lipids (Sanchez *et al.*, 2012). Recently it has been demonstrated that RLs fit into plant lipid‐based membrane models and are located near the lipid phosphate group of the phospholipid bilayers, near phospholipid glycerol backbones (Monnier *et al.*, 2019). RL insertion inside the lipid bilayer does not strongly affect lipid dynamics but the nature of the phytosterols could influence the effect of RLs on plant plasma membrane destabilization. These subtle changes in lipid dynamics could be linked with plant defence induction (Monnier *et al.*, 2019). Interestingly, synthetic molecules derived from the RL structure are also known to induce plant immunity. Synthetic rhamnolipid bolaforms (SRBs), composed of two rhamnoses separated by a fatty acid chain (Obounou Akong *et al*., 2015), trigger an immune response in *Arabidopsis* that varies according to fatty acid chain length (Luzuriaga‐Loaiza *et al.*, 2018). Ac‐RLs and Alk‐RLs, only differing from natural RLs by the terminal group of the carbon chain (with a methyl for Alk‐RLs and a carboxylic acid for Ac‐RLs), induce ROS production in *Arabidopsis* (Nasir *et al.*, 2017). Interestingly, these synthetic RLs are able to interact with membrane lipids, suggesting that perception of these molecules could involve a lipid‐driven process (Luzuriaga‐Loaiza *et al.*, 2018; Nasir *et al.*, 2017). Alk‐RLs were more favourably inserted into model membranes and induced a higher response than Ac‐RL, suggesting that differences in the biological activity of these molecules could be linked to their amphiphilic nature and their capacity to interact with the membrane (Nasir *et al.*, 2017). The synthetic 3‐tetradecylamino‐*tert*‐butyl‐*N*‐tetradecylpropionamidine (diC_14_) lipid is known to induce TLR4‐dependent mechanisms in mammals. It has also been studied for its eliciting properties in *Arabidopsis* and interestingly the plant defence response induced by the molecule is independent from CERK1 (Cambiagno *et al.*, 2015). The chain length of the lipid influences the immune response in *Arabidopsis*. diC_14_ and diC_16_ induce defence‐related gene expression in this plant, but diC_16_ leads to weaker responses. diC_14_ pretreatment triggers *Arabidopsis* resistance against *P. syringae*, unlike diC_16_ (Cambiagno *et al.*, 2015). It was therefore hypothesized that the interaction of diC_14_ with plant plasma membrane lipids may alter the organization, compartmentalization or composition of this membrane to somehow boost the activity of the plant defence system (Cambiagno *et al.*, 2015).

#### Polysaccharide‐derived IPs

Some studies have shown that the LysM domain of CERK1 has very weak binding affinity to chitosan (Iizasa *et al.*, 2010; Petutschnig *et al.*, 2010). Moreover, defence genes are up‐regulated by chitosan, both in wild‐type *Arabidopsis* and chitin‐insensitive *cerk1* mutant, demonstrating that chitosan is perceived through a CERK1‐independent pathway (Povero *et al.*, 2011). In addition, chitosan can interact with phospholipids bilayers (Fang *et al.*, 2001). As proposed for several IPs, chitosan could induce membrane structure modifications, stimulating plant immunity (Iriti and Varoni, 2015). As for ergosterol, it was also demonstrated that chitosan directly affects plasma membrane H^+^‐ATPase, giving rise to a possible link between chitosan‐triggered plant innate immunity and its putative impact on the structural organization of lipid rafts from the plant plasma membrane (Amborabé *et al.*, 2008; Rossard *et al.*, 2010).

## Conclusion

Apoplastic IPs are diverse in their molecular nature: some are kingdom‐specific or even specific to species, while others are present in several kingdoms, such as chitin, which is found in fungi, bacteria and arthropods. The majority of apoplastic IPs characterized to date are perceived by plants at the plasma membrane through PRRs. Interestingly, more and more studies are also suggesting a new perception system based on direct sensing through membrane lipids without the involvement of specific proteinaceous PRRs. This sensing system monitors membrane perturbations and is driven by amphiphilic compounds or toxin‐like compounds. Interestingly, some IPs, such as NLPs, elicitins or chitosan, can be perceived through direct interaction with PRRs and/or by lipid‐mediated mechanisms. In addition, IPs known or suspected to be perceived through membrane lipids are only from microbial origin. Many studies are still required to understand how IPs are sensed by plants and it seems that a large variety of processes are involved. Further integrative investigations, including biophysical approaches and functional biology on plasma membrane lipids, are required to characterize lipid‐based IP perception and its potential relationship with PRR‐mediated mechanisms. The understanding of IP‐triggered immunity is a first step in the development of new plant breeding strategies. PRR engineering and modification of the lipid composition of the plasma membrane could impact the ability of plants to perceive IPs and are examples of biotechnologies that could be used to optimize plant resistance.
